# PROTOCOL: Risk and protective factors for child sexual abuse and interventions against child sexual abuse: An umbrella review

**DOI:** 10.1002/cl2.70000

**Published:** 2024-11-04

**Authors:** Izabela Zych, Inmaculada Marín‐López

**Affiliations:** ^1^ Department of Psychology Universidad de Cordoba Cordoba Spain

## Abstract

This is the protocol for a Campbell Collaboration systematic review. Our objective is to conduct an umbrella review to synthesize published and unpublished systematic reviews focused on risk and protective factors for child sexual abuse and effectiveness of interventions against child sexual abuse perpetration and victimization. Specific research questions are: (i) what are the risk and protective factors for child sexual abuse victimization, and what are their relative strength and/or magnitude for predicting child sexual abuse victimization? (ii) what are the risk and protective factors for child sexual abuse perpetration, and what are their relative strength and/or magnitude for predicting child sexual abuse perpetration? (iii) are interventions aimed at reducing and/or preventing child sexual abuse effective? (iv) what are the moderators that increase or decrease effectiveness of the interventions? Efforts to decrease child sexual abuse need to be based on research, but more accessible evidence regarding the breadth of risk and protective factors and effectiveness of interventions to reduce child sexual abuse needs to be provided to policymakers. This will be the first umbrella review that comprehensively synthesizes findings of the previous systematic reviews that focus on risk and protective factors for child sexual abuse and interventions to prevent or reduce child sexual abuse. The results will be able to inform enhanced prevention policy and programs, and regulatory measures for specific contexts of child sexual abuse.

## 1 BACKGROUND

### 1.1 The problem, condition, or issue

Child sexual abuse is an extremely harmful type of violence with devastating consequences for individuals and societies that impacts children's mental, physical, and social outcomes (Hailes et al., 2019). Many serious short‐ and long‐term consequences have been confirmed in meta‐analytic reviews, including posttraumatic stress disorder, suicide (Paolucci et al., 2001), depression and Fanxiety (Lindert et al., 2014), problematic sexual behavior (Noll et al., 2019; Paolucci et al., 2001), poor academic performance (Fry et al., 2018) and poor long‐term physical health (Irish et al., 2010). A meta‐review by Nagtegaal and Boonmann (2022) showed that child sexual abuse was related to sexual, health, and psychological problems. Mental health problems are a common long‐term consequence of child sexual abuse (Chen et al., 2010; Guiney et al., 2024; Hailes et al., 2019; Noll, 2021).

Child sexual abuse has been more precisely defined over time as more is understood about its nature, characteristics, and effects. In landmark definitional research, Mathews and Collin‐Vézina (2019) proposed four factors that must be present for an act or experience to be conceptualized as child sexual abuse: (i) the target person is a child, (ii) true consent is absent, (iii) the act is done to seek sexual gratification for the abuser or for another person, and it can be immediate or deferred in time and space, and (iv) the act occurs in the context of a relationship in which there is any type of power inequality (e.g., physical, psychological, relational) used to exploit the child's vulnerability. Recently, the International Classification of Violence Against Children (UNICEF, 2023, pp. 31–32) defined sexual violence against a child as “any deliberate, unwanted and non‐essential sexual act either completed or attempted, that is perpetrated against a child including for exploitative purposes, and that results in or has a high likelihood of resulting in injury, pain or psychological suffering.” It is worth mentioning that different forms of child abuse and neglect tend to co‐occur, and many children can be considered as polyvictims (Debowska et al., 2017).

Even after decades of research that has established evidence‐based primary, secondary, and tertiary intervention approaches, child sexual abuse is still a widespread phenomenon around the world (Contreras Taibo et al., 2020; Finkelhor, 1994; Kloppen et al., 2016; Pan et al., 2021; Pereda, Guilera, Forns, Gómez‐Benito, 2009; Stoltenborgh et al., 2011; Walakira, 2002). Prevalence rates vary among studies by geographical areas. According to meta‐analytic reviews, the worldwide prevalence among females is approximately 24% (Pan et al., 2021), and prevalence rates for males are generally lower (Moody et al., 2018). According to a systematic review by Barth et al. (2013), prevalence rates of child sexual abuse of females range 8‐31% while prevalence rates for males range 3–17%. Child sexual victimization can occur in early childhood (Pereda, 2023), and most research suggests that it peaks around adolescence (Black et al., 2001). Most research shows a much higher proportion of male perpetrators then female perpetrators (Hunt, 2006; Seto et al., 2015) with the highest proportion of perpetrators being adults who are known to the child (parents/caregivers in children's homes, institutional caregivers and other known adults), together with a substantial proportion of adolescent offenders, also mostly known to the child victim (Mathews et al., 2024). Thus, gender differences in victimization and offending require special attention.

Child sexual abuse should be considered on a non‐contact‐to‐contact continuum (Fergusson et al., 2013). Non‐contact sexual abuse can involve sexual jokes, exhibitionism, voyeurism, exposure to pornography, unwanted sexting, stalking, or grooming. Contact sexual abuse can involve forced sexual touching and forced intercourse. According to a systematic review and meta‐analysis focused on prevalence rates of child sexual abuse worldwide (Barth et al., 2013), non‐contact abuse is much more frequent than contact abuse, and its most severe form—forced intercourse (17%, 6%, and 3% of overall prevalence rates for males and 31%, 13%, and 9% of overall prevalence rates for females, respectively). These acts can happen within families and in other settings including different institutions such as out‐of‐home care, religious organizations, sporting associations, and educational facilities (Australian Government, 2013). Widespread use of electronic devices has now provided a relatively new context for child sexual abuse with an important proportion of these acts facilitated by technologies (Finkelhor et al., 2024). Severity of child sexual abuse can also be considered taking into account a combination of factors such as type (non‐contact to contact), chronicity (e.g., an isolated incident vs. repeated over time), and relation to the child (e.g., stranger vs. family member) with examples of the most severe cases described as a repeated penetrative acts perpetrated by a family member or other individuals (Lyons & Romano, 2019).

There is a growing concern and awareness regarding different forms of child maltreatment and the number of studies on the topic has substantially increased in the past decade (Finch et al., 2021). Nevertheless, child sexual abuse is still present and prevalent around the world (Gewirtz‐Meydan & Finkelhor, 2020; Pereda et al., 2024; Pereda, Guilera, Forns, Gómez‐Benito, 2009), and there are pressing gaps in knowledge that need to be addressed (Russell et al., 2020). Among them, it remains necessary to synthesize knowledge of risk and protective factors for child sexual abuse and to understand which interventions are effective in preventing or reducing child sexual abuse.

Even though preventing child sexual abuse is one of the top priorities worldwide (European Commission, 2020; The U.S. Department of Homeland Security, 2020) including the eradication of violence against children among the UN Sustainable Development Goals, knowledge about its risk and protective factors and effectiveness of interventions and moderators that increase or decrease the effectiveness of interventions against child sexual abuse is fragmented. There is a large number of interventions against child sexual abuse, and research syntheses conducted to date have focused on different outcomes such as knowledge of the problem, protective behaviors, anxiety and fear, and disclosure (e.g., Walsh et al., 2015). Currently available systematic reviews and meta‐analyses are valuable to understand different parts of the problem, but it is necessary to synthesize knowledge using a more comprehensive and broader approach to provide a global panorama of the field.

### 1.2 Risk and protective factors for child sexual abuse

This umbrella review will include published and unpublished systematic reviews focused on risk and protective factors for child sexual abuse. The review will include both victim and perpetrator‐related risk factors, which we define as the variables related to demographics, mental health, psychological, social, physical, and other individual or contextual characteristics that could increase the likelihood of child sexual abuse victimization or perpetration, respectively. Victim and perpetrator‐related protective factors are variables related to all the previously mentioned characteristics that, in turn, could decrease the likelihood of child sexual abuse victimization and perpetration. Protective factors can be divided into promotive (protective) factors and buffering protective factors. Promotive factors are directly related to the outcome while buffering factors reduce the negative outcomes of present risk factors (Lösel & Farrington, 2012; Seto et al., 2023). Both risk and protective factors for the onset of child sexual abuse, but also for recidivism or revictimization are going to be included. Specific examples of risk and protective factors could include but are not limited to: family‐related factors, victim disability, exposure to child pornography, history of child sexual abuse, trauma, level of empathy, neuropsychological functioning, and mental health. Risk and protective factors may, therefore, occur at individual, family, community, or wider societal levels. Risk and protective factors can differ according to the setting (e.g., family vs. institution), victim‐perpetrator relationship (e.g., family vs. strangers) and severity of child sexual abuse (e.g., non‐contact vs. rape).

### 1.3 The outcome

This umbrella review focuses on intrafamilial and extrafamilial child sexual abuse victimization and perpetration online and offline. This can be either self‐reported, other reported, or measured through official reports. Any type of child sexual abuse along the continuum from non‐contact (e.g., exposure) to contact (e.g., forced sexual touching) and forced intercourse is eligible. It focuses on abuse of a child (under 18 years old) without a true consent through an act of a sexual nature and under an imbalance of power. Following UNICEF (2023), an act is considered violence against a child if it is deliberate, unwanted, non‐essential, and harmful. “Child” is defined as a person under 18 given that, in most countries, adulthood is defined as being over 18 years old and this is a frequent cut‐off in international classifications of violence against children (see UNICEF, 2023) and scientific literature on child sexual abuse (see, e.g., Assink et al., 2019; Barth et al., 2013; Walsh et al., 2015; Zhang et al., 2021).

### 1.4 How risk and protective factors might be linked to the outcome

Several theories have been proposed to understand risk and protective factors for child sexual abuse. Most of these theories focus on understanding the perpetration of sexual offenses against children, while fewer explain why some children may be more vulnerable to victimization.

According to a review of theories focused on child sexual abuse offending conducted by Dangerfield et al. (2020), child sexual abuse can be explained by focusing on single factors and by multi‐factor theoretical approaches. Focusing on specific risk and protective factors, a systematic review and meta‐analysis conducted by Whitaker et al. (2008) divided these factors into family‐related (e.g., harsh discipline, abusive parents), externalizing behaviors (e.g., antisocial personality), internalizing behaviors (e.g., anxiety, low self‐esteem), social difficulties (e.g., low social skills, loneliness), and sexual behaviors (e.g., deviant sexual interest in children). All these factors can increase individual's vulnerability towards deviant sexual behavior that could be further explained by multi‐factor models described below. Child abuse victimization can also be a risk factor for perpetration, although it was found that child physical abuse in males (not females) was related to more sex offences, while there was no significant relation between child sexual victimization and future sex offences (Widom & Massey, 2015). There is also evidence on previous victimization being a risk factor for revictimization which could be related to the perpetrator factors, vulnerability of the victim and guardian factors (Contreras et al., 2022).

Risk and protective factors can differ across settings, with some specific features and actions that can be present to a different degree in institutions, but that are not applicable in other setting such as homes. According to Australia's Royal Commission, these can be related to the organizational culture, awareness, and reaction by managers and staff, child protection policy, staff, and student education about sexual abuse, reporting mechanisms, access to children by known offenders, private access to children, prioritizing children's welfare and external oversight (Mathews, 2017). As stated by Ward and Fortune (2016) risk factors can be predictive, but they have limitations because they can be best conceptualized as clusters of items that do not necessarily cohere. Thus, more complex models with different levels of analysis that focus on different factors are useful.

Finkelhor's (1994) Precondition Model is probably the most extended and one of the first multi‐factor theoretical approaches used to explain child sexual abuse. This model is based on the routine activity theory (Cohen & Felson, 1979), according to which violence is a result of a coexistence of a motivated perpetrator, a suitable target, and a lack of social control. According to Finkelhor (1984), there are four preconditions that need to be present for child sexual abuse to occur. One precondition is a high motivation to commit a sexual offense which is a result of one or more of the following: (i) emotional congruence where perpetrators believe that the act could satisfy their emotional needs, (ii) sexual arousal toward a child, and (iii) an existence of a blockage of other possible sexual activities. This is combined with another precondition where the perpetrator experiences disinhibition, overcoming their own restraint regarding sexual offending against children. A motivated and disinhibited perpetrator also needs to overcome external barriers (the fourth precondition) such as limiting supervision by a guardian and resistance of the child. Taking into account the Precondition Model, risk and protective factors could work to increase or decrease the likelihood of each precondition. Some of these factors could refer to the perpetrator (e.g., sexual arousal towards a child or high disinhibition that increase the risk of offending), and some could refer to the victim (e.g., high parental supervision or better strategies to report could be protective).

According to Quadripartite Model (Hall & Hirschman, 1991), there are four factors that underly sexual aggression towards a child including: (i) sexual arousal towards children, (ii) beliefs that justify sexual aggression against children, (iii) Affective dyscontrol (aberrant ways of regulating emotions), and (iv) personality disorders. This model highlights that there can be different types of sexual perpetrators against children depending on which of the four factors is more prominent in each case. Thus, risk and protective factors can coincide with the four components of the Quadripartite Model or they can increase or decrease the likelihood of these components (e.g., there are factors that can increase or decrease the likelihood of having a personality disorder).

The Integrated Theory proposed by Marshall and Barbaree (1990) posits that individuals can be more likely to commit sexual offenses against children because of their vulnerabilities in adolescence. After adverse childhood experiences, vulnerable individuals may not have the requisite skills to satisfy their sexual needs during adolescence in a socially acceptable way. Thus, sexual needs can become linked to aggression. Sexual offending can be a result of a combination of this vulnerability with situational factors such as strain, substance use, or sexual arousal. If this sexual offense is reinforced, it tends to be repeated. More recently, Marshall and Marshall (2017) suggested that insecure attachment to caregivers that can also guide future relationships across the lifespan could be the most important basis of an individual's vulnerability. Thus, risk and protective factors could be related to vulnerability (e.g., adverse childhood experiences), situational factors (e.g., substance use), and reinforcement mechanisms (e.g., sexual satisfaction). Based on the Integrated Theory, Smallbone et al. (2008) focused even more on situational factors, claiming that child sexual abuse is a result of complex interactions among biological, developmental, ecosystemic, and situational factors.

After critically reviewing previous theories, Ward and Siegert (2002) proposed the Pathways Model to understand child sexual abuse. According to this model, child sexual abuse can be explained through different possible pathways that combine developmental influences, dysfunctional mechanisms, and situational opportunities. Psychological mechanisms that explain child sexual abuse offending may be related to: (i) intimacy and impaired social skills that could be derived from insecure attachment and expectation of rejection, (ii) distorted sexual scripts that could include inappropriate partners, behaviors, or contexts, (iii) emotional dysregulation where individuals find it difficult to control their emotions in service of their goals, and (iv) cognitive distortions that refer to problematic beliefs and attitudes regarding victims' mental states and behaviors or moral appraisal of the offense and a combination of these mechanisms. The Pathways Model recognizes that there are multiple different pathways to offending involving several possibly inter‐related mechanisms, including different risk and protective factors (e.g., sexual abuse in childhood could be a risk factor for distorted sexual scripts in adolescence and adulthood). Each set of mechanisms is involved in sexual offending, but one is hypothesized to be primary throughout the rest of the systems committing human agency and its related contexts.

In sum, there are several models that proport to explain child sexual abuse, and these models focus on different individual, interpersonal, and contextual risk and protective factors. Other theoretical approaches focus on sexual offending in general including, for example, Seto's (2019) Motivation‐Facilitation Model or Ward's and Beech's (2006) Integrated Theory of Sexual Offending. According to Seto's Motivation‐Facilitation Model, there are some traits that increase the motivation to commit sexual offences, such as paraphilia, high sex drive, and intense effort to find a partner. These motivation factors need to be combined with factors that facilitate sexual offending including trait factors (e.g., antisocial personality), or situational factors (e.g., intoxication). Thus, there must be a motivation to offend that is combined with opportunities that facilitate offending. According to Ward's and Beech's (2006) Integrated Theory of Sexual Offending, sexual offending is a result of an interaction between the factors that affect brain development including evolution, genes neurobiology, and ecological factors focused on circumstances and environment. Additionally, in her Compositional Explanatory Theory of Pedophilia, Gannon (2021) suggests that sexual interest or preference for prepubescent children is a combination of different biological, environmental, and psychological variables, acknowledging at the same time that pedophilia is a risk factor that is important, but not sufficient to cause child sexual abuse. All these models highlight the importance of risk and protective factors that could explain offending. Even though theories and research on the topic have been fruitful, it is still necessary to establish which are the most important factors that could increase the risk or protect against child sexual abuse based on a comprehensive synthesis of previous findings that can be achieved through an umbrella review. Thus, this umbrella review focuses on risk and protective factors for victimization and perpetration, but it is acknowledged that factors might only be relevant for one and not the other, and they can function differently (e.g., female sex can be a risk factor for victimization while male sex can be a risk factor for perpetration).

### 1.5 Interventions against child sexual abuse

#### 1.5.1 The intervention

This umbrella review focuses on any type of intervention that aims at decreasing child sexual abuse. Interventions may be conducted in any setting (e.g., institutions, clinical settings, families) by any type of facilitators (e.g., psychologists, teachers, parents) and focus on any type of child sexual abuse on the spectrum from noncontact to contact acts, including but not limited to forced intercourse. Interventions can be primary, secondary, or tertiary and can be aimed at reducing victimization or perpetration (or both). Interventions may be delivered directly to potential victims, perpetrators, or other agents (e.g., institutional leaders, police, teachers, other children, or adolescents) and especially to guardians (e.g., parents). Interventions may focus on broader contexts in which child sexual abuse occurs (e.g., improving online or offline safety). They can be implemented at the individual, family, institutional, community, or societal level. Interventions can focus on general population (primary prevention), vulnerable groups (secondary prevention), or victims and perpetrators who have already been involved in or experienced child sexual abuse (tertiary prevention). For perpetration, interventions can be implemented in community setting and in custody, with some effect size differences between the two (Koehler & Lösel, 2024). Thus, this umbrella review is conducted with a broad focus on programs or policies against child sexual abuse. Examples include, but are not limited to, individual‐level and society‐level interventions such as community treatment packages for sex offenders (Craissati et al., 2002), biological treatments such as hormonal agents (Maletzky & Field, 2003), behavioral treatments including aversion and reconditioning (Marshall & Barbaree, 1988), programs that increase help‐seeking skills in adolescent girls who could become potential victims (Sinclair et al., 2013), social marketing campaigns (Horsfall et al., 2010) and teaching school children about different topics related to child sexual abuse (Lu et al., 2023; Madrid et al., 2020).

From the public health perspective (see Kemshall & Moulden, 2016), primary prevention usually targets the whole community to raise awareness and change attitudes regarding child sexual abuse (e.g., posters and slogans focused on myths related to child sexual abuse), secondary level interventions focus on people at risk (e.g., a helpline for people concerned about their sexual attraction to children) while tertiary prevention could focus on helping the victims and reducing their risk of revictimization (e.g., a widely publicized helpline for victims). There is also a growing body of knowledge regarding promising comprehensive sexual education programs that are now being conducted in K‐12 (Schneider & Hirsch, 2020). Legislative and regulatory mechanisms such as mandatory reporting, working with children checks, reportable conduct schemes, and helplines, among others, could also be considered together with regulatory measures in child and youth serving organizations (e.g., situational crime prevention, child safety standards legislation schemes).

Interventions focused on primary, secondary, and/or tertiary prevention are eligible if it is explicitly stated that they aim to decrease or prevent child sexual abuse victimization and/or perpetration. Interventions not aimed at decreasing child sexual abuse, but focused on other objectives (e.g., decreasing mental‐health consequences of child sexual abuse for the victims) will be excluded. Reviews with multiple objectives are eligible as long as at least one objective aligns with the current inclusion criteria and studies aimed to decrease child sexual abuse are synthesized separately from the studies that do not align with our inclusion criteria.

#### 1.5.2 How the intervention might work

Taking into account different theories that explain sexual offending against children, interventions could focus on decreasing risks and increasing protective factors. The theoretical link between these factors and effective interventions aimed to reduce child sexual abuse can be explained by several models. The Good Lives Model (Ward et al., 2007), for example, states that sexual offences are committed when people do not have the abilities to fulfill their needs in a socially and personally desirable way. Thus, interventions should help offenders achieve “good lives” by analyzing goods to be achieved together with personal strengths and the environment, and focus on factors that are needed to achieve those goods. According to the Risk‐Need‐Responsivity model (Andrews & Bonta, 2010) the intensity of the intervention needs to be based on the risk of offending, and criminogenic needs (factors directly related to, such as disinhibition) should be addressed using the responsivity principle that adjusts the intervention to the person's abilities and characteristics.

Regarding Finkelhor's Precondition Model (Finkelhor, 1984), interventions could decrease motivation to sexually offend against children by modifying perpetrator's beliefs about possible emotional satisfaction derived from the act, reducing their sexual arousal through behavioral treatment, and enhancing skills and knowledge making it possible for the perpetrators to satisfy their sexual needs in a socially desirable way. Also, disinhibition could be decreased, for example, through cognitive‐behavioral interventions. Interventions focused on reducing victimization could also increase external barriers, for example, by encouraging parental monitoring and supervision by caregivers, increasing surveillance in institutions, training child serving workforces in identifying, reporting, and responding to child sexual abuse and teaching children about their rights to bodily integrity and autonomy, and strategies for help seeking. Based on Quadripartite Model (Hall and Hirschman, 1991), treatments could aim at decreasing sexual arousal towards children through directed masturbation, decrease their dysfunctional beliefs, improve desirable emotion regulation strategies, and enhance adaptative strategies and cognitions to improve functioning and interpersonal relationships among individuals with personality disorders (Ward & Siegert, 2002).

Based on the Integrated Theory (Marshall & Barbaree, 1990), interventions could focus on decreasing childhood and adolescent vulnerability (e.g., improving parenting skills to promote secure attachment) and situational factors (e.g., decreasing substance use). Interventions can also focus on situational and structural drivers of sexual violence considering the individual‐situation interaction (Smallbone et al., 2008). Based on the Pathway Model (Ward & Siegert, 2002), interventions could potentially decrease the risk and increase protective factors for each pathway that leads to different mechanisms that underlay child sexual abuse (e.g., by increasing secure attachment, increasing emotional regulation, and decreasing antisocial cognitions). Also, based on Motivation‐Facilitation Model (Seto, 2019), interventions could decrease both motivation factors (e.g., by targeting paraphilia through directed masturbation) and facilitation factors (e.g., by targeting substance use). In relation to Ward's and Beech's (2006) Integrated Theory of Sexual Offending, improving brain development and ecological factors could also result in less sexual offending against children. At the same time, if risk factors are not direct causes, theories can be helpful to interpret how they are linked to child sexual abuse. Interventions can also work differently depending on the context (e.g., institutions vs. families) or severity of child sexual abuse. In general, taking into account that most theories are multifactorial, interventions need to address multiple risk and protective factors at the same time.

### 1.6 Why it is important to do this review

There are several existing systematic reviews and meta‐analyses that have focused on child sexual abuse. However, none of these has specifically focused on a comprehensive synthesis of knowledge derived from systematic review on any possible risk and protective factor for victimization and offending and interventions against child sexual abuse. To date, no registered PROSPERO protocols on the topic have been located. Conducting a single systematic review with such a broad aim would be highly difficult and unfeasible due to the large number of studies and variables. Thus, an umbrella review that would integrate all available systematic reviews on the topic is needed and timely to synthesize the breadth of evidence. An umbrella review may also identify areas that have not yet been synthesized and inform future review efforts.

Among the previous umbrella reviews focused on child sexual abuse, Hailes et al. (2019) synthesized meta‐analyses on a wide range of long‐term consequences of child sexual abuse. They included 19 meta‐analyses with 28 long‐term outcomes of child sexual abuse, but they did not focus on risk and protective factors or interventions. Thus, Hailes et al.'s (2019) review is useful to understand one part of the field, but its aims were different from the current umbrella systematic review. Another umbrella review conducted by Massullo et al. (2023) focused on prevalence and definitions of child maltreatment, abuse, and neglect. They included studies on child sexual abuse, but only in relation to definitions and prevalence rates.

Many previous systematic reviews focused on specific risk and protective factors for child sexual abuse. Among them, Assink et al. (2019) synthesized 72 studies with 765 risk factors for child sexual victimization which were further classified into 35 domains. These domains included child's sex, possible physical or mental conditions, parental history of child abuse victimization, intimate partner violence among parents, parental relationship problems, substance use, mental or physical problems, low parental education, low parental care, low parenting competence, family history of antisocial behavior, prior child abuse, problems in family functioning, social isolation, family structure (non‐nuclear with and without a stepfather), low family SES, and religious family affiliation.

Augarde and Rydon‐Grange (2022) systematically reviewed 86 studies on female perpetrators of child sexual abuse including a broad range of related variables. Babchishin et al. (2015) analyzed a broad range of characteristics of online versus offline child sexual perpetrators, but their systematic review only included primary research with identifiable sample of online or mixed (online and offline) perpetrators. Laird et al. (2020) focused on factors related to child sexual exploitation (a subtype of child sexual abuse). They meta‐analyzed 37 studies including 52 demographic and psychosocial variables related to child sexual exploitation. Turner and Rettenberger (2020) conducted a systematic review of research on neuropsychological factors related to child sexual abusers versus other perpetrators. They included 25 primary studies on this specific topic. Thus, several research syntheses have been conducted regarding risk or protective factors against child sexual abuse but the previous studies focused on specific subfields within the topic, and a more comprehensive synthesis of existing systematic reviews is still required.

There are several systematic reviews focused on different specific contexts or outcomes of interventions to prevent or reduce child sexual abuse. Among them, Russell et al. (2020) conducted a systematic review of interventions against child sexual abuse in developing countries. They narratively described the methodology and design of the included studies, characteristics of participants, and included a brief description of the content of the included interventions and outcome measures. Another systematic review on the effectiveness of interventions against child sexual abuse was published by Walsh et al. (2015). This seminal Campbell/Cochrane review analyzed school‐based education programs against child sexual abuse. Similarly, Zhang et al. (2021) meta‐analyzed school‐based interventions against child sexual abuse in China. In a Campbell systematic review, Schmucker and Lösel (2017) meta‐analyzed the effectiveness of interventions to reduce recidivism among sex perpetrators in general but did not focus on children specifically. Hanson et al. (2009) conducted a meta‐analysis on the principles of correctional treatments for sex perpetrators in general. Thus, specific geographic areas such as developing countries (e.g., Russell et al., 2020) or specific contexts such as schools (e.g., Walsh et al., 2015) have been covered by previous systematic reviews, but it is still necessary to conduct a comprehensive umbrella review that would make it possible to obtain a global panorama of the field.

In the past decade, many systematic reviews have also focused on various topics related to child sexual abuse that differ from the current review including treatments or therapies for victims (e.g., Benuto & O'Donohue, 2015; Lu et al., 2020; Parker & Turner, 2013), for perpetrators (e.g., Gronnerod et al., 2015; McPhail & Olver, 2020; Sousa et al., 2022), and caregivers (e.g., St‐Amand et al., 2022). Other reviews were conducted on specific types of child sexual abuse, such as child exploitation material offending (e.g., Eggins et al., 2021). All these reviews and/or meta‐analyses provide important data on child sexual abuse, or sex offending in general, but none of the previous reviews has included a comprehensive umbrella analysis of risk and protective factors, and interventions to prevent or reduce child sexual abuse.

Child sexual abuse is extremely harmful for the abused children and for the societies in general. Decreasing this problem is among the top policy priorities worldwide (European Commission, 2020; The U.S. Department of Homeland Security, 2020). Efforts to decrease child sexual abuse need to be based on research, but more accessible evidence regarding the breadth of risk and protective factors and effectiveness of interventions to reduce child sexual abuse needs to be provided to policymakers. This will be the first umbrella review that comprehensively synthesizes findings of the previous systematic reviews that focus on risk and protective factors for child sexual abuse and interventions to prevent or reduce child sexual abuse. The results will be able to inform enhanced prevention policy and programs, and regulatory measures for specific contexts of child sexual abuse perpetration (e.g., within child and youth serving organizations). Eradication of violence against children has been included in the UN Sustainable Development Goals and this review will contribute useful information to inform these efforts. Thus, policymakers will have the opportunity to make evidence‐informed decisions about intervention strategies based on a global panorama that has not been available before. The achievement of the objectives of this umbrella review, individually and collectively, can contribute knowledge to inform policy developments, practice reforms, and practitioner training.

## 2 OBJECTIVES

This umbrella review aims to synthesize published and unpublished systematic reviews focused on risk and protective factors for child sexual abuse and effectiveness of interventions against child sexual abuse perpetration and victimization.

Specific research questions are:

1. What are the risk and protective factors for child sexual abuse victimization, and what are their relative strength and/or magnitude for predicting child sexual abuse victimization?
2. What are the risk and protective factors for child sexual abuse perpetration, and what are their relative strength and/or magnitude for predicting child sexual abuse perpetration?
3. Are interventions aimed at reducing and/or preventing child sexual abuse effective?
4. What are the moderators that increase or decrease effectiveness of the interventions?

## 3 METHODS

### 3.1 Criteria for considering reviews for inclusion

#### 3.1.1 Types of studies

This umbrella review will include systematic reviews only. Following Wilson et al. (2024), we define a systematic review as a review study that is based on systematic searches of published and unpublished studies, that has explicit inclusion and exclusion criteria, an established protocol to extract and code the results of the primary studies, and a way to assess the quality of the included studies. Systematic reviews that do and do not include a meta‐analysis are eligible. Systematic reviews of quantitative research will be included, and systematic reviews of qualitative research will be excluded because they need a different research synthesis methodology. Mixed‐methods systematic reviews that include an eligible quantitative part will be included. Other types of literature reviews (e.g., non‐systematic narrative reviews) and editorial materials will be excluded. Both published and unpublished studies will be eligible. Also, any publication type, including theses, conference proceedings, journal articles, among others, are eligible if a systematic review is reported. Empty reviews (i.e., where no eligible studies were located) are also eligible. Protocols for completed reviews will be linked to the publication of the completed review and counted as one study and multiple documents. Protocols for incomplete reviews will be included if their stated inclusion criteria align with the inclusion criteria for this review. These will be included in an “Ongoing studies” reference list in the final review.

##### Objectives 1 and 2: Risk and protective factors

Systematic reviews of risk and protective factors for child sexual abuse perpetration and victimization will be included if these provide a synthesis of primary studies using correlational, cross‐sectional, and preferably longitudinal designs. Studies included in eligible reviews can be correlational, where the risk/protective factor and outcome are continuous variables, or they can utilize a design that compares groups with and without the risk/protective factor. Reviews will be included if they provide effect sizes on the relation between any possible risk and/or protective factor and child sexual abuse. If available, only bivariate results will be included. Multipredictor models will only be included if bivariate results are not available. Bivariate results are preferred over other models results because multipredictor analyses are inconsistent among studies, and different numbers and types of variables controlled for make it difficult to interpret and compare the findings.

##### Objectives 3 and 4: Interventions

Intervention reviews will be included if these synthesize at least one randomized controlled trial or a quasi‐experiment with a robust design and provide an effect size on effectiveness of the intervention. Robust designs refer to reviews of quasi‐experiments if they are based on nonrandomized experimental versus control group with a pre‐ and a posttest measure which include a control group of some description. Any comparison condition is eligible (e.g., waiting list, treatment‐as‐usual, other treatments). Reviews that include only uncontrolled intervention studies with one group pre‐ and posttest only will be excluded. Reviews of only qualitative evidence will be excluded.

#### 3.1.2 Types of participants

##### Objectives 1 and 2: Risk and protective factors

Individual, relational, contextual, or any other risk and protective factor included in the reviews need to be measured in relation to perpetrators or victims of child sexual abuse. We define victims of child sexual abuse as a person under 18 years old who has been a target of any type of sexual abuse. We define perpetrators of child sexual abuse as any person who perpetrated any type of sexual abuse of a minor. Nevertheless, sexual dating violence (i.e., perpetrated by a dating partner) will be excluded as this is considered a specific phenomenon with a whole field of research (Rodriguez‐deArriba et al., 2024) and specific underlying mechanisms different from child sexual abuse. Regarding participants' characteristics, there will be no other restrictions in terms of study location or any other characteristic of the participants (e.g., age, sex, gender, ethnicity). Perpetrators or victims of child sexual abuse need to be compared to non‐perpetrators or non‐victims of child sexual abuse in terms of risk and protective factors or different levels/frequencies of child sexual abuse need to be related to the risk and protective factors. Studies that compare child sexual abuse offenders to other offenders (or other non‐general populations) or child sexual victims to other victims (or other non‐general populations) will also be included. Also, studies that compare different types of sexual offenders against children (e.g., online and offline) will be included. Findings regarding each comparison group will be described separately and it will be explicitly acknowledged that the comparison group needs to be taken into account while interpreting the findings. At the same time, this heterogeneity of the comparison groups could be useful to capture some factors that could be specific to sexual offenders against children versus other offenders (see, e.g., Gannon, 2021). Studies that only include adults, even if retrospective (e.g., adults who were victims of child sexual abuse) are going to be excluded.

##### Objectives 3 and 4: Interventions

Intervention participants can be any type of population (potential victims, perpetrators, caregivers, practitioners, law enforcement, etc.). Again, there will be no other restrictions regarding study location or any characteristic of the participants (e.g., age, sex, gender, ethnicity).

#### 3.1.3 Types of outcome measures

##### Objectives 1 and 2: Risk and protective factors

For risk and protective factors, outcomes will be child sexual abuse victimization or perpetration. We define child sexual abuse to be an act of a sexual nature where a target is a child, true consent is absent, and there is an imbalance of power. Child sexual abuse can be self‐reported by victims, perpetrators, other‐reported (e.g., by teachers, caregivers), and based on official reports (i.e., arrests and convictions, including recidivism). Any type of sexual violence against a child is eligible. If child sexual abuse is a part of a broader construct (e.g., child abuse in general, sex offending in general) and cannot be distinguished as a separate variable, reviews will be excluded.

##### Objectives 3 and 4: Interventions

For interventions, studies are not going to be included or excluded based on the measured outcomes. The actual child sexual abuse (self‐reports, other reports, and official reports: arrests and convictions, including recidivism) will be the main outcome. Outcomes considered as indicators of the risk of child sexual abuse described in the included reviews will also be eligible. These may include, but will not be limited to, knowledge about child sexual abuse, protective behaviors, disclosure, institutional safety‐related policy and practice, and risk assessment scores. Recidivism rates are also eligible in interventions aimed at perpetrators. Any other outcome, including outcomes related to the consequences of child sexual abuse, can be included (e.g., posttraumatic stress in victims). This is because some systematic reviews focused on interventions against child sexual abuse report secondary outcomes related to consequences (e.g., Walsh et al., 2015). Again, reviews that do not provide specific information regarding child sexual abuse but rather include it as a part of a broader construct (e.g., sex offending in general) will be excluded.

#### 3.1.4 Types of risk and protective factors (Objective 1)

A risk factor for child sexual abuse is defined as any factor that could increase the probability of child sexual abuse perpetration or victimization. A protective factor is defined as any factor that could decrease the probability of child sexual abuse perpetration or victimization. In longitudinal studies, risk and protective factors precede child sexual abuse perpetration or victimization, and in cross‐sectional projects, these factors are treated as independent variables and theoretically conceptualized as risk and protective factors. Risk and protective factors for perpetration could include, but are not limited to, personality disorders, social skills, and sexual arousal towards a child. Risk and protective factors for victimization could include, but are not limited to parental supervision, disability, and help‐seeking skills. Risk and protective factors can be measured through any type of measure including self‐reports, other‐reports (e.g., caregivers), and official records, among others.

#### 3.1.5 Types of intervention (Objective 2)

This umbrella review will include systematic reviews focused on any type of intervention that aims at decreasing child sexual abuse perpetration and/or victimization, and this needs to be explicitly stated by the authors. If aims are not explicitly stated, the focus of the interventions will be analyzed, and reviews of interventions with a clear focus on decreasing child sexual abuse will be included. As defined by Eggins et al. (2021) and Mazerolle et al. (2021), interventions are programs, strategies, tools, campaigns, directives, or any other activities intended to modify or target a problem. Interventions for primary, secondary, and tertiary prevention are eligible. These include interventions aiming to prevent or reduce recidivism. Interventions are expected to focus on decreasing risk factors and increasing protective factors. They are expected to be aimed at children, potential perpetrators, caregivers, institutions, law enforcement officers, and the general population. Examples of interventions could include increasing strategies and skills for help seeking of the potential victims or cognitive‐behavioral or behavioral psychological treatments for the perpetrators. Interventions focused on the consequences of child sexual abuse only (e.g., improving mental health in victims) are excluded from this umbrella review. Also, interventions focused on related topics that do not explicitly aim at decreasing child sexual abuse are going to be excluded (e.g., interventions aiming at training practitioners to report child abuse by Walsh et al., 2022).

#### 3.1.6 Timing of outcome assessment(s)

##### Objectives 1 and 2: Risk and protective factors

Ideally, child sexual abuse should be measured after the measurement of the risk and protective factors through longitudinal research, preferably with several waves of data collection. Nevertheless, cross‐sectional studies where the outcome is measured at the same time as the theoretically suggested risk and protective factors are eligible. Studies where child sexual abuse is measured as a predictor of other outcomes considered its consequences will be excluded; either where child sexual abuse is measured before the outcomes or concurrently with theoretically conceptualized consequences. This means that studies where child sexual abuse is not considered as an outcome, but only as a risk factor for other outcomes (e.g., depression later in life) will be excluded. Also, case‐control studies where victims or perpetrators of child sexual abuse are compared to non‐victims or non‐perpetrators of child sexual abuse are going to be included.

##### Objectives 3 and 4: Interventions

No restrictions regarding the timing of outcome assessment are going to be applied. Most of the interventions are expected to include a posttest immediately after the intervention, and some could include a follow‐up. Any length of the follow‐up is eligible.

#### 3.1.7 Types of settings

##### Objectives 1 and 2: Risk and protective factors

There will be no restrictions regarding the contexts or settings for the risk and protective factors. This means that individual and contextual factors are eligible. Among them, individual factors such as personality, history of child sexual abuse victimization, and disability are of interest, together with family factors and factors related to peers, school, and other institutions. Other factors are also eligible. There will be no restrictions regarding the publication language or geographical area. Searches will be conducted in English, but studies in any language not understood by the authors (other than English, Spanish, and Polish) will be translated by Google Translate. In case of doubt regarding the translation, international colleagues will be asked for help.

Following the umbrella review by Wilson et al. (2024), searches will be restricted to systematic reviews published after 1990. This is because it is highly unlikely to find systematic reviews performed with robust methodology before 1990 and systematic reviews published after 1990 are expected to cover the topics and literature included in older reviews.

##### Objectives 3 and 4: Interventions

This umbrella review will not place any restrictions regarding settings where eligible interventions take place. Interventions are expected to be conducted in different institutions including schools and prisons. Also, there may be some interventions that take place at the individual level through cognitive‐behavioral counseling (e.g., by psychologists in their office), family‐based interventions, and broader campaigns or policies at the population level. Once again, there will be no restrictions regarding publication languages or location, but only reviews published from 1990 onwards will be eligible.

### 3.2 Advisory group

This umbrella review will include an advisory group of leading experts in the field of child sexual abuse from nine different countries and six different continents. Advisory group includes:

- Lorena Contreras Taibo (Facultad de Psicología, Universidad Diego Portales, Chile).
- R. Karl Hanson (Department of Psychology, Carleton University, Ottawa, Canada).
- Friedrich Lösel (Institute of Psychology, Friedrich‐Alexander‐University Erlangen‐Nuremberg, Germany and Institute of Criminology, University of Cambridge, UK).
- Bernadette Madrid (Philippine General Hospital, University of Philippines Manila, Philippines).
- Noemí Pereda (Departament de Psicologia Clínica i Psicobiologia, Universitat de Barcelona, Spain).
- Michael Seto (Institute of Mental Health Research at The Royal, University of Ottawa, Canada).
- Heather Turner (Crimes against Children Research Center, University of New Hampshire, USA).
- Eddy Joshua Walakira (Makerere University, School of Social Sciences, Uganda).
- Kerryann Walsh (School of Early Childhood and Inclusive Education, Creative Industries, Education and Social Justice, Queensland University of Technology, Australia).
- Tony Ward (School of Psychology, Victoria University of Wellington, New Zealand).

Members of the advisory group have been asked to provide their feedback on the draft versions of the protocol and the review. Members reviewed the theoretical part of this umbrella review and were asked to pay special attention to the methods section and advise the review authors regarding search terms and sources, inclusion and exclusion criteria, and the best way to code and synthesize data. They have also been asked to provide any additional published and unpublished systematic reviews that may have been missed from the draft set of the included studies and distribute this request to their colleagues.

### 3.3 Search methods for identification of reviews

#### 3.3.1 Electronic searches

Comprehensive searches will be performed through specific search terms aiming to identify all the eligible systematic reviews and meta‐analyses. Electronic searches will be performed the following databases:

- Australian Criminology Database (CINCH) (Informit).
- Criminal Justice Abstracts (EBSCOhost).
- PsycINFO (Ovid).
- PsycEXTRA (Ovid).
- MEDLINE (Ovid).
- Joanna Briggs Institute EBP Database (Ovid).
- Epistemonikos (https://www.epistemonikos.org/en/).
- Book Citation Index—Social Sciences & Humanities (Web of Science).
- Conference Proceedings Citation Index—Social Sciences & Humanities (Web of Science).
- Emerging Sources Citation Index (Web of Science).
- Korean Journal Database (Web of Science).
- SciELO Citation Index (Web of Science).
- Social Science Citation Index (Web of Science).
- Campbell Systematic Reviews (Wiley).
- Cochrane Database of Systematic Reviews (https://www.cochranelibrary.com/).
- SCOPUS (Elsevier).
- Dissertations & Theses Global (ProQuest).
- International Bibliography of the Social Sciences (ProQuest).
- Social Services Abstracts (ProQuest).
- Sociological Abstracts (ProQuest).

Information including dates of each search, search location and an exact search syntax will be recorded for each database. Searches will be performed by an information retrieval specialist in titles, abstracts, keywords and subject/indexing terms.

Drawing on the previous reviews in the field (Hine et al., 2023; Schmucker & Lösel, 2017; Walsh et al., 2015; Wilson et al., 2024), our own experience, suggestions sent by the advisory board members and the Department of Homeland Security, the following search terms and structure have been developed, through a combination of search terms using Boolean operators “AND” and “OR” and proximity operators.

Terms related to population will be:

adolesc* OR babies OR baby OR boy OR boys or child* OR girl OR girls OR infanc* OR infant* OR juvenil* OR minor OR minors OR neonatal OR preadolesc* OR pre‐adult* OR pre‐school* OR preschool* OR prepubesc* OR preteen* OR pubescen* OR pupil OR pupils OR school‐age* OR “school age*” OR student* OR teen* OR toddler* OR underage* OR youngs* OR youth*

Terms related to problem/topic will be:

(sex* adj3 (abus* OR aggress* OR assault* OR crime? OR crimin* OR coerc* OR commer* OR exploit* OR harass* OR maltreat* OR mal‐treat* OR mistreat* OR mis‐treat* OR nonconsen* OR non‐consen* OR offenc* OR offend* OR offens* OR perpetrat* OR solicit* OR traffick* OR victim* OR violen* OR unwanted)

OR

(child* adj3 abus*)

OR

(CSAM OR sex doll* or grooming OR incest* OR molest* OR paedophil* OR paraphil* OR pederast* OR pedophil* OR rape? OR rapist? OR fondl* OR forc* sex* OR “commercial sex*” OR “sex work*” OR exhibitionis* OR “revenge porn*” OR sextort* OR voyeur*)

OR

(aggravated OR nonconsen* OR non‐consen*) adj2 sext*).

OR

(cyber OR digital OR internet* OR livestream* OR live stream* OR mobile online OR on‐line OR technology‐assisted OR technology‐facilitated) adj3 (abus* OR sex*).

OR

(forc* adj2 intercourse)

OR

(image‐based adj4 abus*).

Terms related to study design will be:

“systematic review” OR (systematic* adj3 review*) OR “meta analys* OR “gap map*” OR meta‐analy* OR metaanaly* OR meta‐review* OR metasynthes* OR meta‐synthes* OR “scoping review*” OR “umbrella review*” OR (quantitative* adj3 (overview* OR review* OR synthes*))

Supporting Information S1: Appendix 1 provides the syntax for a search for PsycInfo and Medline databases. Searches will be restricted to publications after 1990.

#### 3.3.2 Searching other resources

Hand searches will be performed in the following leading journals in the field:

- *Aggression and Violent Behavior*
- *Child Abuse and Neglect*
- *Child Maltreatment*
- *Clinical Psychology Review*
- *Criminal Behaviour and Mental Health*
- *Criminal Justice Behavior*
- *European Journal of Criminology*
- *Journal of Child Sexual Abuse*
- *Journal of Developmental and Life Course Criminology*
- *Journal of Experimental Criminology*
- *Journal of Interpersonal Violence*
- *Journal of Offender Rehabilitation*
- *Journal of Offender Therapy and Comparative Criminology*
- *Journal of Sexual Aggression*
- *Journal of Traumatic Stress*
- *Psychology, Crime & Law*
- *Sexual Abuse*
- *Sexual Offending: Theory, Research, and Prevention*
- *Trauma, Violence and Abuse*

Journals will be searched using their websites by screening all published titles and abstracts including issues in the past 2 years from the systematic search date (including possible articles in press).

Searches for gray literature (i.e., unpublished or published in nontraditional channels) will be captured by some of the search sources listed above (i.e., Dissertations and Theses, PsycEXTRA) and will also be sought by searching the following sources:

- 3ie: https://www.3ieimpact.org/
- Australian Institute of Criminology: https://www.aic.gov.au/
- Association for the Treatment of Sexual Abusers (ATSA): https://www.atsa.com/
- Crime Reduction Toolkit by College of Policing: https://www.college.police.uk/research/crime-reduction-toolkit
- CrimeSolutions: https://crimesolutions.ojp.gov
- the Department of Homeland Security: https://www.dhs.gov/
- International Society for the Prevention of Child Abuse and Neglect: https://ispcan.org/
- the National Organisation for the Treatment of Abuse (NOTA): https://www.nota.co.uk/
- RAND: https://www.rand.org
- Public Safety Canada: https://www.publicsafety.gc.ca
- Save the Children: https://www.savethechildren.org/
- the UNESCO: https://www.unesco.org
- UNICEF: https://www.unicef.org
- What Works to Prevent Violence Against Women and Girls: https://ww2preventvawg.org/
- Youth Endowment Fund Toolkit: https://youthendowmentfund.org.uk/toolkit/

Also, these organizations will be contacted and asked to provide any relevant documents. Furthermore, Advisory group members and corresponding authors of the included studies will be asked to provide any additional published or unpublished systematic reviews and circulate the request among their colleagues. Reference lists of the included studies will also be screened for potentially eligible studies.

### 3.4 Data collection and analysis

#### 3.4.1 Criteria for determination of independent findings

As in any umbrella review, findings are not expected to be independent. This is mainly because different systematic reviews are expected to include a partially or fully overlapping set of studies given that they may be focused on similar topics. In some cases, there may be multiple publications derived from the same project or original systematic reviews and updated systematic reviews, with the latter including all the studies from the former. Thus, primary studies will be carefully compared, and if all the studies overlap, manuscripts will be treated as complementary reports of the same systematic review. In other cases, there may be two or more manuscripts with a partially overlapping set of studies. These will be described separately, and the percentage of overlap will be reported. A corrected covered area index (Pieper et al., 2014) will be calculated for the reviews focused on overlapping topics (e.g., school‐based interventions against child sexual abuse victimization) to estimate the overlap. Considering that the findings are not expected to be independent, no meta‐analysis will be performed.

#### 3.4.2 Selection of reviews

Searches will be performed in electronic databases and located studies will be exported into Covidence software where possible. Potentially eligible additional manuscripts located through hand‐searches of journals, sent by experts and authors of the included studies, and located on the websites of different agencies will also be added.

Duplicates will be removed in Endnote by the information retrieval specialist and the remaining titles and abstracts will be screened first. If a study is potentially eligible based on title and/or abstract, the full‐text manuscript will be saved for full‐text screening. Records will be considered eligible at title/abstract screening stage if they include terms related to child sexual abuse and terms related to systematic reviews and do not state explicitly that they are only synthesizing qualitative evidence. Screening will be performed by both authors of the current umbrella review independently, and a *κ* agreement rate will be calculated. Discrepancies will be solved through discussion and consensus.

Full texts will also be screened by both authors of this umbrella review using an eligibility screening form (see Table 1). An agreement rate (*κ*) will be calculated, and discrepancies will be solved through discussion and consensus. A PRISMA flowchart will be built to show the study selection process.

**Table 1:** Eligibility screening form.

Eligibility criterion	Decision
Is this a systematic review?	Yes No Cannot tell If no, stop here
Does this systematic review focus on child sexual abuse (victims are children under 18)?	Yes No Cannot tell If no, stop here
Relationship between risk/protective factors and child sexual abuse/violence is described or there is an intervention aimed at preventing or reducing child sexual abuse	Yes No Cannot tell If no, stop here
There is a comparison group for risk/protective factors (e.g., non‐abused children, varying intensity of abuse) or an eligible intervention	Yes No Cannot tell If no, stop here

#### 3.4.3 Data extraction and management

Data will be extracted and coded in Covidence independently by both authors of this umbrella review, and an agreement rate will be calculated. Disagreements will be solved by discussion and consensus. Two coding sheets have been developed, one for risk and protective factors and one for interventions (see Supporting Information S1: Appendix 1). These coding sheets are similar, but each includes specific information focused on one of the two subtopics:

1. Reviews focused on risk and protective factors for child sexual abuse will be coded on the following domains: study information, participants (number, age, sex, ethnic‐cultural background, location, socioeconomic status, recruitment strategy, and settings), design and procedure (list of risk and protective factors analyzed, age of exposure, source of risk/protective factor measure, focus on offending vs. victimization, types of child sexual abuse included, source of child sexual abuse measure and design) and results (type, effect sizes per analyzed relation, number of primary studies and participants per analysis, moderator and subgroup analyzes and authors' conclusion).
2. Reviews focused on interventions against child sexual abuse will be coded including: study information, participants (number, age, sex, ethnic‐cultural background, location, SES, recruitment strategy and setting), design and procedure (intervention name and description, the intervention aims, providers and targets, intervention prevention level, types of child sexual abuse targeted, included outcomes, design, assignment to experimental vs. control group, timing of outcomes measured, follow‐up, source of child sexual abuse measure, intervention settings, duration, and components), results (type of results, effect sizes per outcome, number of primary studies and participants per outcome, moderators, subgroups, components, and narrative conclusions).

#### 3.4.4 Assessment of methodological quality of included reviews

Quality will be assessed through AMSTAR 2 (A Measurement Tool to Assess systematic Reviews).^1^ AMSTAR 2 is an instrument specifically designed to assess the methodological quality of systematic reviews (Shea et al., 2017). It includes 16 items focused on: (i) the inclusion of the PICO components in the research questions and inclusion criteria, (ii) an explicit statement about the review methods being established beforehand and a justification of deviations from the protocol, (iii) an explanation of the selection of the study designs, (iv) an existence of a comprehensive literature search strategy, (v) study selection in duplicate, (vi) data extraction in duplicate, (vii) a list of excluded studies and a justification of their exclusion, (viii) a detailed description of the included studies, (ix) an adequate assessment of the risk of bias, (x) a report of the sources of funding of the included studies, (xi) appropriate methods for the meta‐analysis (if performed), (xii) an assessment of the impact of the risk of bias on the results of the meta‐analysis (if performed), (xiii) consideration of the risk of bias when interpreting the results, (xiv) an explanation and discussion of heterogeneity in the results, (xv) an adequate investigation of publication bias and its impact, and (xvi) a report of a possible conflict of interest.

AMSTAR 2 will be slightly modified when assessing the quality of the studies focused on risk and protective factors for child sexual abuse victimization and perpetration given that the tool was developed to assess the quality of reviews of intervention studies. Thus, PICO components (criterion 1) will refer to population, risk and protective factors (corresponding to interventions), comparison group, and child sexual abuse victimization or perpetration (corresponding to outcome). Regarding the justification of the study designs (criterion 3), we will focus on the justification of designs for studying risk and protective factors (i.e., longitudinal, cross‐sectional, and case‐control). Based on AMSTAR tool (Shea et al., 2007), for quality assessment (criterion 9), checklists and scoring tools will be accepted if results for each study are provided (e.g., low, high). Regarding the impact of the risk of bias (criteria 12 and 13), this will be based on the assessment from criterion 9. All the other AMSTAR 2 criteria can be directly applied to study objectives 1 and 2.

AMSTAR 2 will be applied by both authors of this umbrella review independently, and discrepancies will be solved by discussion and agreement. Quality of the primary studies included in each review will not be assessed, but the tool used and the quality assessment outcomes reported by each included systematic review will be reported.

#### 3.4.5 Dealing with missing data

This umbrella review will synthesize information available in the included systematic reviews. This means that we do not intend to obtain data from the primary studies if this data is missing. Some reviews may meet all systematic review criteria, but do not report effect sizes or other information needed for the syntheses. For those reviews, authors will be contacted for missing data and if they cannot provide it, these reviews will be included in the summary tables that focus on study characteristics but not in quantitative syntheses of meta‐analyses or single effect sizes. AMSTAR ratings will not be applied for such reviews.

If reviews focused on broader topics (e.g., child abuse in general, sex offending) are located through our searches but they do not focus on or include data on child sexual abuse interventions or risk and protective factors, authors of such reviews will be contacted and asked to provide specific data regarding child sexual abuse. For example, it is possible that a broader review focused on child abuse, in general, includes child sexual abuse variables that are not described or analyzed separately. In such cases, authors will be contacted and asked if they do have and could provide separate data regarding sexual abuse only. If this is not possible, the review will be excluded. Thus, reviews will not be excluded based on missing data, but some reviews that do not meet our inclusion criteria (and focus on similar broader topics) would be treated as indicators of possibly unpublished existing data that will be requested and included if provided.

#### 3.4.6 Data synthesis

For both objectives, data will be synthesized through summary tables. Also, forest plots will be produced for similar groups of interventions and outcomes, but without calculating an overall effect size. For example, separate tables and forest plots could be produced for school‐based versus community‐based interventions, for interventions focused on offending, victimization, or both, or for outcomes such as knowledge, skills, and disclosure. If the included reviews report different effect sizes, they will be transformed into the most commonly used effect (e.g., Cohen's *d*). A meta‐analysis will not be performed because we expect to find a certain degree of overlap among the primary studies included in each systematic review (see Pollock et al., 2022), and it would be difficult to accurately capture the strength of the dependencies and to calculate the proper covariances of effect sizes. Also, if different systematic reviews focus on different types of interventions, it does not seem to be meaningful to produce a unique effect size. For the systematic reviews that combine eligible (e.g., RCTs) and non‐eligible (e.g., uncontrolled pre‐test posttest only designs without a control group), if available, only the results of the eligible primary studies will be synthesized in this umbrella review. If not available, overall conclusions will be mentioned in the summary tables, but they will not be included in the forest plots with quantitative findings. For interventions where two treatments against child sexual abuse are compared, conclusions will also be described in the summary table, and separate forest plots will be produced.

#### 3.4.7 Subgroup analysis and investigation of heterogeneity

Given that this umbrella review will not include a meta‐analysis, we do not expect to perform a subgroup analysis or check for heterogeneity. However, Objective 4 focuses on moderators that might increase or decrease the effectiveness of the interventions. Thus, if the included systematic reviews do include a subgroup or moderator analyses, these will be reported and synthesized in the current umbrella review in‐text and summary tables. Subgroup analyses could include, but will not be limited to sex, age groups, ethnic‐cultural groups, mainstream versus special education students.

#### 3.4.8 Treatment of qualitative research

This umbrella review focuses on systematic reviews of quantitative empirical research. Thus, qualitative studies are not going to be included in this review.

## ROLES AND RESPONSIBILITIES

This systematic review will be conducted by a team from the University of Cordoba (Spain).

- Content: Zych and Marín‐López
- Systematic review methods: Zych and Marín‐López
- Statistical analysis: Zych and Marín‐López
- Information retrieval: Zych and Marín‐López

Izabela Zych is a Professor of Psychology at the University of Cordoba and has broad experience and expertise in conducting systematic reviews and meta‐analyses focused on different topics related to violence. She has successfully led and published similar studies, including papers in the leading journals in the field. She is a member of Campbell Collaboration Crime and Justice Group Steering Committee.

Inmaculada Marín López is an assistant profesor in Psychology at the University of Cordoba. She holds a PhD in Psychology. She has participated in numerous national and regional research projects, published several articles in high impact journals, as well as book chapters and several presentations at international conferences. Her research lines focus on the prevention of violence against children, including victimization. She has experience in conducting systematic reviews.

## SOURCES OF SUPPORT

This study was supported by the DHS Science and Technology Directorate and the Five Research and Development (5RD) Countering Violent Extremism Network, awarded to the study authors via the Campbell Collaboration.

## DECLARATIONS OF INTEREST

The authors declare that there are no conflicts of interests. However, one of the review authors has an internal role within the Campbell Collaboration Crime and Justice Group. Specifically, Izabela Zych is a member of the Campbell Collaboration Crime and Justice Group Steering Committee, including the Campbell/DHS Counter‐Terrorism Systematic Review Advisory Board. Thus, Izabela Zych is not involved in any editorial or internal Campbell Collaboration communications about this review.

## PLANS FOR UPDATING THE REVIEW

We plan to produce an updated version of this review every 5 years. The lead author (Izabela Zych) will be in charge of coordinating the revised versions.

## Supporting information

Supporting information.

## Data Availability

Data will be available upon request.

## Footnotes

1
https://amstar.ca
