# Supplementary material for: PROTOCOL: Risk and protective factors for child sexual abuse and interventions against child sexual abuse: An umbrella review
Source: Campbell Syst Rev. 2024 Nov 4;20(4):e70000. doi: 10.1002/cl2.70000 (PMC11534711; doi:10.1002/cl2.70000)
Supplement: Supplementary file 1 — Supporting information. [file CL2-20-e70000-s001.docx]

**Appendix 1. Example search strategy**

Database/Platform: APA PsycInfo <1806 to July Week 1 2024>

Search Date: 6 July 2024

--------------------------------------------------------------------------------

1 (neonatal birth 1 mo or infancy 2 23 mo or preschool age 2 5 yrs or school age 6 12 yrs or adolescence 13 17 yrs).ag. (810789)

2 (adolesc* or babies or baby or boy or boys or child* or girl or girls or infanc* or infant* or juvenil* or minor or minors or preadolesc* or pre-adult* or pre-school* or preschool* or prepubesc* or preteen* or pubescen* or pupil or pupils or school-age* or "school age*" or student* or teen* or toddler* or underage* or youngs* or youth*).mp. (1935590)

3 or/1-2 [population] (2013906)

4 sexual abuse/ or sex offences/ (23779)

5 (sex* adj3 (abus* or aggress* or assault* or crime? or crimin* or coerc* or commer* or exploit* or harass* or maltreat* or mal-treat* or mistreat* or mis-treat* or nonconsen* or non-consen* or offenc* or offend* or offens* or perpetrat* or solicit* or traffick* or victim* or violen* or unwanted)).mp. (74211)

6 child abuse/ or (child* adj3 abus*).mp. (52697)

7 (CSAM or sex doll*).mp. (63)

8 incest/ or (groom* or incest* or molest*).mp. (13481)

9 pedophilia/ or (paedophil* or paraphil* or pederast* or pedophil*).mp. (6800)

10 rape/ or (rape? or rapist? or fondl* or "forc* sex*").mp. (13996)

11 sex work/ or ("commercial sex*" or prostitute* or prostitution or "sex work*").mp. (8358)

12 ((aggravated or nonconsen* or non-consen*) adj2 sext*).mp. (32)

13 ((cyber* or digital* or internet* or livestream* or "live stream*" or mobile* or online or on-line or technology-assisted or technology-facilitated) adj3 (abus* or sex*)).mp. (3518)

14 exhibitionism/ or exhibitionis*.mp. (1397)

15 (forc* adj2 intercourse).mp. (120)

16 (image-based adj4 abus*).mp. (90)

17 ("revenge porn*" or sextort*).mp. (137)

18 voyeurism/ or voyeur*.mp. (685)

19 or/4-18 [problem/topic] (127877)

20 "systematic review"/ or (systematic adj3 review*).mp. (59825)

21 meta analysis/ or ("gap map*" or meta-analy* or metaanaly* or meta-review* or metasynthes* or meta-synthes* or "scoping review*" or "umbrella review*").mp. (65735)

22 (quantitative adj3 (overview* or review* or synthes*)).mp. (3202)

23 (cinahl or cochrane or embase or medline or pubmed or searched).ab. (53405)

24 or/20-23 [study designs] (124559)

25 3 and 19 and 24 (2195)

26 limit 25 to yr="1990 -Current" (2186)

**Appendix 2. Coding scheme**

**RISK AND PROTECTIVE FACTORS FOR CHILD SEXUAL ABUSE**

- - - 1. **Coder and coding date:**
      2. **Study information**
- Authors: names
- Authors: countries of affiliation
- Publication year
- Study title
- Document language
- Document type: journal article, Campbell/Cochrane review, PhD thesis, book, book chapter, agency/government report, unpublished document, other (specify)
- Funding received: yes/no (if yes, specify the funding agency name)
- Conflict of interest: yes/no (if yes, describe)
- Objective:

**3. Methodology**

**3.1. Participants**

- total number of participants (all primary studied included) and min-max
- Age (min-max)
- Sex: percentage of female (min-max)
- Ethnic-cultural background (percentage min-max): Caucasian, African American, Latin American, Asian American, other (specify)
- Locations of the primary studies (specify countries or geographic areas depending on the available information)
- socioeconomic status: percentage of studies with low, medium, high, mixed as reported by the review
- recruitment strategy: percentage of studies using convenience sampling, random selection from a population, cohort, other (specify)
- settings where participants were recruited (percentage of studies per setting, e.g., prisons, schools)

**3.2. Design and procedure**

- List of risk and protective factors analyzed
- Age of exposure to each risk and protective factor (range) and age of sexual abuse (range)
- Length of time since sexual abuse
- Source of risk and protective factor measure (percentages): self-reports, other-reports (specify), official records, other (specify)
- Included studies focused on (percentages): perpetration, victimization
- Comparison group: general population, other (specify)
- Types of child sexual abuse included (percentages per offending and victimization): contact (specify), non-contact (specify), forced intercourse (specify), other (specify)
- Source of child sexual abuse measure (percentages): self-reports, other-reports (specify), official records, other (specify)
- Design (percentages): cross-sectional, longitudinal prospective, other (describe)

**4. Results**

- Type of results: meta-analysis, systematic review without meta-analysis, other (specify)
- Meta-analysis: yes/no, if yes code:
  1. Overall effect size (and CI) for each meta-analyzed relation between risk/protective factor and each outcome
  2. Number of primary studies and participants for each meta-analyzed relation between risk/protective factor and each outcome
  3. Moderation analysis: yes/no, if yes, code effect sizes (and CIs) per moderator and each risk/protective factor-outcome relation analyzed, and number of primary studies
  4. Subgroup analysis: yes/no, if yes, code effect sizes (and CIs) per group and each risk/protective factor-outcome relation analyzed, and number of primary studies
- Meta-analysis: yes/no, if yes, code method: Fixed-effect, Random effects, other (specify)
- If no meta-analysis, code:
  1. Effect size (and CI) for each primary study regarding the relation between risk/protective factors and each analyzed outcome, and number of participants
  2. Number of participants for each meta-analyzed relation between risk/protective factor and each outcome, and number of participants
  3. Effect size (and CI) per moderator in each primary study and each risk/protective factor-outcome relation analyzed, and number of participants
  4. Effect size (and CI) peer subgroup in each primary study each risk/protective factor-outcome relation analyzed, and number of participants
- Vote counting? Yes/no
- Autors´ narrative main conclusion:

**INTERVENTIONS AGAINST CHILD SEXUAL ABUSE**

1. **Coder and coding date:**

2. **Study information**

- Authors: names
- Authors: countries of affiliation
- Publication year
- Study title
- Document language
- Document type: journal article, Campbell/Cochrane review, PhD thesis, book, book chapter, agency/government report, unpublished document, other (specify)
- Funding received: yes/no (if yes, specify the funding agency name)
- Conflict of interest: yes/no (if yes, describe)
- Objective:

**3. Methodology**

**3.1. Participants**

- Total number of participants (all primary studied included), and min-max per experimental vs. control groups
- Age (min-max)
- Sex: percentage of female (min-max)
- Ethnic-cultural background (percentage min-max): Caucasian, African American, Latin American, Asian American, other (specify)
- Locations of the primary studies (specify countries or geographic areas depending on the available information)
- socioeconomic status: percentage of studies with low, medium, high, mixed
- recruitment strategy: percentage of studies using convenience sampling, random selection from a population, other (specify)
- settings where participants were recruited (percentage of studies per setting, e.g., prisons, schools)

**3.2. Design and procedure**

- Intervention name(s) and short description(s):
- Interventions aimed at (percentages) reducing: offending, victimization
- Provider of the intervention: researchers, NGO, business organization, multiple (specify, e.g., researchers train teachers, teachers provide intervention)
- Target of the intervention (percentages): perpetrators, victims, schoolchildren, teachers, families, children-other (specify), institutional personnel (specify), law enforcement, general population, other (specify)
- Intervention prevention level (percentages): primary (general population), secondary (individuals at risk), tertiary (perpetrators or victims)
- Types of child sexual abuse targeted (percentages per offending and victimization): contact (specify), non-contact (specify), forced intercourse (specify), other (specify)
- Included outcomes (percentage of studies per outcome):
- Design (percentage): randomized controlled trial, quasi-experiment, other (specify)
- Assignment to experimental vs. control group (percentage): random-individuals, random-clusters (specify), convenience (specify), other (specify)
- Type of control/comparison treatment (percentage): treatment as usual, waiting list, another CSA intervention, other (specify)
- Outcomes measured at (percentage): pre-test and post-test in experimental and control groups, pre-test and post-test in experimental group only (no controls), post-test only in experimental and control groups, post-test in experimental group only (no controls), other (specify)
- Analysis (percentage): intention-to-treat, per protocol
- Presence of a follow-up (percentage), if yes, specify duration
- Source of child sexual abuse measure (percentages): self-reports, other-reports (specify), official records, other (specify)
- Intervention settings (percentage): school, prison, community (specify), other
- Intervention duration: min-max
- Intervention components (percentage of studies per component)

**4. Results**

- Type of results: meta-analysis, systematic review without meta-analysis, other (specify)
- Meta-analysis: yes/no, if yes code:
  1. Overall effect size (and CI) for each meta-analyzed outcome
  2. Number of primary studies per each meta-analyzed outcome
  3. Number of participants for each meta-analyzed outcome
  4. Moderation analysis: yes/no, if yes, code effect sizes (and CIs) per moderator and number of primary studies
  5. Subgroup analysis: yes/no, if yes, code effect sizes (and CIs) per group and number of primary studies
  6. Component analysis: yes/no, if yes, code effect sizes (and CIs) per component and number of primary studies
- Meta-analysis: yes/no, if yes, code method: Fixed-effect, Random effects, other (specify)
- If meta-analysis, code how multiple outcomes were treated: multilevel multivariate, combined, independent, other (specify)
- If no meta-analysis, code:
  1. Overall effect size (and CI) for each primary study outcome and number of participants
  2. Moderation analysis: yes/no, if yes, code the effect size (and CI) per moderator in each primary study and number of participants
  3. Subgroup analysis: yes/no, if yes, code effect sizes (and CIs) per group in each primary study and number of participants
  4. Component analysis: yes/no if yes, code effect sizes (and CIs) per component in each primary study and number of participants
- Vote counting? Yes/no
- Autors´ narrative main conclusion:
